# Heart failure with preserved ejection fraction: present status and future directions

**DOI:** 10.1038/s12276-019-0323-2

**Published:** 2019-12-19

**Authors:** Somy Yoon, Gwang Hyeon Eom

**Affiliations:** Department of Pharmacology, Chonnam National University Medical School, Hwasun, 58128 Republic of Korea

**Keywords:** Translational research, Heart failure

## Abstract

The clinical importance of heart failure with preserved ejection fraction (HFpEF) has recently become apparent. HFpEF refers to heart failure (HF) symptoms with normal or near-normal cardiac function on echocardiography. Common clinical features of HFpEF include diastolic dysfunction, reduced compliance, and ventricular hypokinesia. HFpEF differs from the better-known HF with reduced ejection fraction (HFrEF). Despite having a “preserved ejection fraction,” patients with HFpEF have symptoms such as shortness of breath, excessive tiredness, and limited exercise capability. Furthermore, the mortality rate and cumulative survival rate are as severe in HFpEF as they are in HFrEF. While beta-blockers and renin-angiotensin-aldosterone system modulators can improve the survival rate in HFrEF, no known therapeutic agents show similar effectiveness in HFpEF. Researchers have examined molecular events in the development of HFpEF using small and middle-sized animal models. This review discusses HFpEF with regard to etiology and clinical features and introduces the use of mouse and other animal models of human HFpEF.

## Cardiology: Reviewing a heart disease that hides

The condition known as heart failure with preserved ejection fraction (HFpEF), in which the fraction of blood pumped from the heart by each heartbeat remains near normal is an increasing cause of illness and death. Available treatments are unsatisfactory. Heart functions also appears normal or near normal in echocardiography imaging, but patients suffer some typical symptoms of heart failure including shortness of breath, excessive tiredness, and reduced ability to exercise. Gwang Hyeon Eom and Somy Yoon at Chonnam National University in Gwangju, South Korea, review understanding of the causes and clinical features of HFpEF, and the development of animal models to aid research into the condition. Studies in rodents are revealing molecular level cellular changes that may be involved in causing HFpEF. The insights gained will hopefully lead to more effective treatments.

## Heart failure

The heart supplies oxygen and nutrients to the body through the blood circulation. Rhythmic cardiac motion is required to achieve this function. Cardiac activity can roughly be divided into two phases: diastole (or relaxation) and systole (or contraction)^1^. During diastole, the ventricle rapidly increases in volume, with an abrupt decline in intracardiac pressure^2^. When ventricular pressure becomes equal to atrial pressure, the valve between the atrium and ventricle opens. At that moment, blood from a large vein fills the ventricle in early diastole; then, forceful contraction of the atrium moves a small amount of blood into the ventricle. Systole follows diastole. After filling, the ventricle begins to contract. When the contractile pressure exceeds that in a large artery, blood in the ventricle is pushed into the artery. Normal performance in both diastole and systole is required for normal cardiac function^2^.

Heart failure (HF) implies an inadequate blood supply^3^ and is exemplified by systolic HF^4^. The major characteristic of systolic HF is decreased contractile function^3,4^. A patient with HF symptoms and an ejection fraction <40% on echocardiography is diagnosed with systolic HF. Systolic HF is known as HF with reduced ejection fraction (HFrEF)^3,4^. The most common cause of HFrEF is a loss of effective left ventricular myocardium due to myocardial infarction, which is an ischemic event^5^. Reduced circulation triggers compensatory mechanisms^6^. Decreased perfusion in the kidney results in activation of the renin-angiotensin-aldosterone system (RAAS)^7^. At the same time, increased residual blood in the chamber at end-systole stresses the ventricular wall; this promotes neurohormonal activation and leads to eccentric heart remodeling^8^. Because of its central role in the development of HFrEF, targeted therapy against the RAAS (i.e., the use of an angiotensin-converting enzyme inhibitor, type 1 angiotensin receptor blocker, or aldosterone antagonist) is effective in HFrEF patients^3,4^. Beta-adrenoceptor regulators are standard therapies for HFrEF, as these agents significantly reduce cardiac burden and automaticity^3,4^.

Many patients with typical HF symptoms, such as shortness of breath, limited exercise capacity, or cough, have a normal ejection fraction (>50%) on echocardiography, indicating that systolic function is normal. These patients are diagnosed with HF with preserved ejection fraction (HFpEF). HFrEF refers to systolic HF, but HFpEF does not imply diastolic HF.

## Heart failure with a preserved ejection fraction

**Fig. 1:**
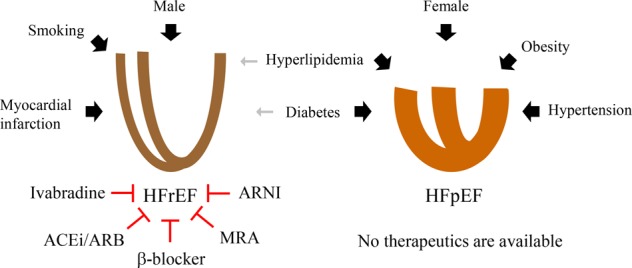
Comorbidity associated with each type of heart failure. HFrEF or systolic heart failure is more common in males. Well-known underlying risk factors include smoking and myocardial death due to infarction. However, HFpEF is more common in females. Comorbidities include obesity, hypertension, hyperlipidemia, and diabetes mellitus. While beta-blockers, ACEi/ARB agents, ARNI, ivabradine, and MRAs reduce mortality in HFrEF, no medications are available for HFpEF. ACEi, angiotensin-converting enzyme inhibitor; ARB, angiotensin II type 1 receptor blocker; ARNI, angiotensin receptor neprilysin inhibitor; HFrEF, heart failure with reduced ejection fraction; HFpEF, heart failure with preserved ejection fraction; MRA, mineralocorticoid receptor antagonist. Arrow widths reflect the association and relevance of hazard ratios. The red blunted lines show effective inhibition of disease progression

The prevalence of HFpEF has increased in the last two decades, and approximately half of all HF patients are diagnosed with HFpEF^9^. HFpEF is more common in women, while HFrEF is more common in men^10^. While many patients have been categorized as having “HFpEF,” their individual characteristics are heterogeneous^11^. Big data analysis of the HFpEF cohort has revealed several risk factors. Shah et al. identified the clinical features of HFpEF: 68% were women, 90% had hypertension, 70% had obesity, 62% had hyperlipidemia, and 52% had diabetes mellitus (DM)^12^. Another research group further supported this finding. Kao et al. clarified the phenotypes of HFpEF: 59% were women, 100% had DM, 84% had hyperlipidemia, and 75% had obesity (>30 kg/m^2^ body mass index) and hypertension^13^. Both studies consistently confirmed common cardiometabolic features: women with hypertension, obesity, DM, or hyperlipidemia (Fig. 1) predominated^12,13^.

Commonly, the systolic function in HFpEF remains within the normal range, but the diastolic function is variable^3,4^. HFpEF is not equivalent to diastolic dysfunction or diastolic HF. Diastolic dysfunction can be defined as a functional defect in relaxation, ventricular filling, or distensibility^14^. Diastolic dysfunction indicates abnormal ventricular mechanical ability. HFpEF is a clinical term used to indicate HF with normal contractile function but without any consideration of diastolic function^4^. Diastolic dysfunction itself is a kind of benign aging process and can be observed in the absence of HF symptoms^15^. Although HFpEF and diastolic heart failure are not synonymous, many clinical features overlap^3,12^. Chronic sustained diastolic dysfunction is a definite risk factor for HFpEF^16^.

Another type of HF with subnormal ejection fraction has recently been described, with 40% < ejection fraction < 50%. This ejection fraction is midway between that of a normal and failing heart and has been defined as HF with mid-range ejection fraction (HFmrEF)^4^. Clinically, HFmrEF shares features of both HFpEF and HFrEF^17^. In the absence of a cohort study for HFmrEF, the management approaches vary. Some clinicians treat HFmrEF and HFrEF similarly, while others treat these as cases of borderline HFpEF^18^. Some experts argue that HFmrEF represents a transition between HFpEF and HFrEF^19^, but the evidence is not convincing. The symptoms and hospitalization patterns resemble those of HFpEF, while clinical features, prognosis, and outcomes of HFmrEF are similar to those of HFrEF^4,20^.

## Pharmacological interventions for heart failure with a preserved ejection fraction

Pharmacological treatment for HFrEF is well established. Beta-adrenoceptor blockers, RAAS inhibitors, and mineralocorticoid receptor blockers can reduce morbidity and mortality in HFrEF patients^3,4^. Large-scale clinical trials have been instituted to identify effective drugs based on the risk factors for HFpEF^21–23^, some of which are ongoing.

The beta (β)-adrenergic system is primarily located in the heart (β1) and smooth muscle (β2). When endogenous catecholamines activate the β1 signaling cascade, the heart contracts more rapidly and forcefully^24^. However, aberrant activation of the neurohormonal system worsens cardiac remodeling in HFrEF, implying that β-adrenergic blockage is beneficial^25^. The β-blockers allow the heart to rest through a significant reduction in cardiac load. Various studies using β-blockers have been conducted using different patient enrollment criteria. However, the overall outcome of β-blocker trials revealed no survival benefit for HFpEF patients^22^. HFmrEF patients showed better survival than HFpEF patients with β-blocker treatment^20^.

The RAAS controls both blood pressure and the circulating volume of blood by regulating vascular tone and sodium reabsorption, respectively^26^. When the baroreceptors in the carotid sinus detect falling blood pressure or when the filtration flow rate in the kidney stimulates the macula densa, the homeostatic system in the body activates the RAAS^27^. The main active component of the RAAS is angiotensin II. Hence, the administration of an angiotensin-converting enzyme inhibitor (ACEi) to block the generation of the active form of angiotensin from angiotensin I, or the use of an angiotensin receptor blocker (ARB) or AT1 receptor blocker, can inhibit HF progression^28^. Although ACEi/ARB agents are effective in HFrEF, clear evidence was not observed in HFpEF^22^.

Aldosterone, an endogenous mineralocorticoid hormone, accelerates myocardial remodeling, especially after an ischemic event. Mineralocorticoid receptor blockers alleviate myocardial fibrosis and extracellular matrix deposition^29^. Pfeffer et al. reported that aldosterone antagonists could be beneficial for HFpEF in the Treatment of Preserved Cardiac Function Heart Failure with an Aldosterone Antagonist (TOPCAT) clinical trial in the United States^30^. Beldhuis et al. warned that the prescription of an aldosterone antagonist should be avoided in HFpEF with decreased renal function^31^. However, cautious interpretation of the TOPCAT study is required. Other than in patients in the United States, aldosterone antagonists have shown no benefits. Furthermore, no positive outcome was found in the analysis of summary data from multiple facilities^30^. More detailed and long-term studies involving different countries or races are needed.

The absence of clear evidence of benefit from β-blockers, RAAS inhibitors, or aldosterone antagonists prompted numerous clinical trials. Physicians chose medicines according to the risk factors for HFpEF to perform these clinical trials. Nitric oxide directly stimulates and relaxes vascular smooth muscle^32^. The use of a nitric oxide donor is the most useful strategy for the reduction in peripheral vascular resistance, i.e., afterload. Redfield et al. performed a trial using isosorbide mononitrate, an oral long-acting nitric oxide derivative, and found that exercise capacity was not improved^23^. The Inorganic Nitrite Delivery to Improve Exercise Capacity in HFpEF (INDIE-HFpEF) clinical trial enrolled additional patients. Disappointingly, inorganic nitrite failed to improve exercise capacity^33^. Further, sildenafil, a phosphodiesterase-5 inhibitor, was evaluated. Redfield et al., in the Phosphodiesterase-5 Inhibition to Improve Clinical Status and Exercise Capacity in Diastolic Heart Failure (RELAX) trial, reported that sildenafil treatment failed to improve exercise capacity or clinical status^34^. Several trials with limited numbers of patients showed promising outcomes with the use of lipid-lowering statins^35^. Confirmation would require a well-designed clinical trial with adequate power. In summary, clearly effective medication treatment for HFpEF remains undefined.

Recently, several therapeutics have been developed for HFrEF. The negative inotropic agent ivabradine was first approved in angina^36^, and its indication has expanded to chronic HFrEF^37,38^. Ivabradine significantly reduced the risk of hospitalization in HF patients who were tolerant to beta-blockers and had a left ventricular ejection fraction of <35% and a heart rate that exceeded 70 beats per minute^38^. A randomized, double-blinded, placebo-controlled trial to assess the effectiveness of ivabradine in HFpEF was completed (EDIFY trial). Unfortunately, the reduction in heart rate by ivabradine failed to improve the clinical outcome of HFpEF^39^.

Angiotensin receptor neprilysin inhibitor (ARNI) is a combination drug that includes conventional angiotensin blockers and neprilysin inhibitors. Although neprilysin degrades several vasoactive peptides, the natriuretic peptide is a notable substrate. Compared with ACEi or ARB, ARNI reduced the rate of cardiovascular death or hospitalization by 20%^40^. A randomized clinical trial on HFpEF (PARAGON-HF; NCT01920711) with ARNI has completed patient enrollment. The PARAGON-HF trial will determine the usefulness of the combination drug ARNI in HFpEF^41^.

It is noteworthy to observe the clinical outcome of the sodium-glucose co-transporter-2 (SGLT2) inhibitor trial. Originally, SGLT2 inhibitors were used for the treatment of type II DM. SGLT2 inhibitors interfere with sodium-glucose-linked co-transporter 2 in renal proximal convoluted tubules, which then reduces glucose reuptake^42^. A notable clinical outcome was observed in the EMPA-REG trial: cardiovascular events were reduced when the SGLT2 inhibitor was combined with conventional treatment regimens^43^. New clinical trials such as DELIVER (NCT03619213), EMPEROR-Preserved (NCT03057951), and PRESERVED-HF (NCT03030235) are ongoing to determine the therapeutic potential of SGLT2 in HFpEF patients^44,45^.

## Research using an experimental rodent model for human heart failure with a preserved ejection fraction

### Model validation: cardiac function measured on echocardiography

Clinically, HFpEF and diastolic HF are not synonymous^46^. A significant proportion of HFpEF patients have normal diastolic function and normal cardiac geometry. However, there are more patients with diastolic dysfunction and ventricular hypertrophy than there are patients without diastolic dysfunction. For this reason, an experimental model of human HFpEF generally requires an investigation of diastolic dysfunction, ventricular hypertrophy, interstitial fibrosis, or exercise intolerance^47^.

Echocardiography is widely utilized to assess diastolic function. Ventricular filling is divided into two phases. Ventricular relaxation in early diastole induces rapid and passive ventricular filling, whereas atrial contraction in late diastole adds a small amount of filling to the ventricle^1^. Doppler images record two prominent peaks, which are the E (ventricular relaxation) and A (atrial contraction) waves. As the E wave primarily reflects diastolic properties, a decreased E wave indicates diastolic dysfunction^48^. A significant amount of residual blood in the atrium due to impaired diastolic function augments the atrial contribution to ventricular filling, demonstrated by an increased A wave^48^. A decreased E/A ratio reflects diastolic dysfunction^48^.

**Fig. 2:**
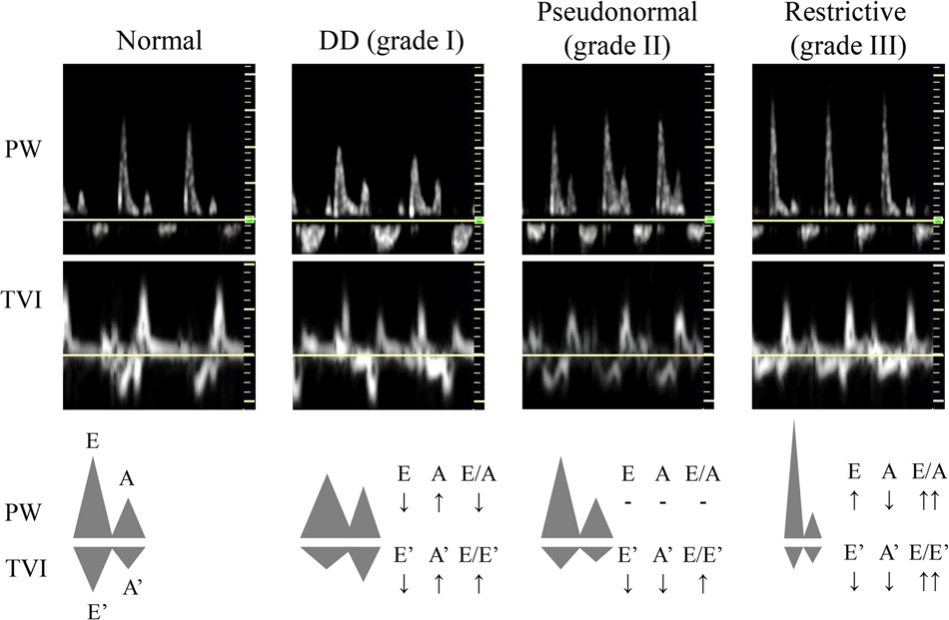
Echocardiographic findings and schematic demonstrations according to the severity of diastolic dysfunction. DD, diastolic dysfunction; PW, pulse wave; TVI, tissue velocity image

When the atrial pressure is further increased, the E wave is increased, but the E/A ratio paradoxically returns to normal. This phenomenon is called a pseudonormal filling pattern^48,49^. For differential diagnosis, the direct measurement of ventricular movement is required. The tissue Doppler imaging mode directly records the mitral valve annulus movement. The two phases of mitral valve motion show waveforms similar to the E and A waves in tissue Doppler mode: the E′ wave represents early diastole, and the A′ wave represents atrial contraction^48^. Because it directly represents ventricular function, the E′ wave is further diminished in the pseudonormal period^48,49^. Therefore, the E/E′ ratio is markedly increased in diastolic dysfunction (Fig. 2). An increased E/E′ ratio is one of the criteria used in the differential diagnosis^4^.

### Model validation: heart failure symptoms with anatomical changes

A rodent model of HFpEF represents cardiac hypertrophy with or without interstitial fibrosis^47^. Experimental cardiac hypertrophy is associated with increased cardiac mass and wall thickness. Simply weighing the intact heart or dividing its weight by total body weight indicates the hypertrophic phenotype^50^. In addition to concentric hypertrophy, which is usual in HFpEF, a heart with normal intracardiac dimensions is commonly observed^51^. Hence, the measurement of wall thickness is mandatory to demonstrate hypertrophy. Many studies have used left ventricular free wall thickness to document hypertrophy. Moreover, cardiac perivascular fibrosis and interstitial fibrosis present as hypertrophy^52^. Because a major component of cardiac fibrosis is the presence of collagen in the extracellular matrix, Masson’s trichrome or Picrosirius red staining can demonstrate its severity^53,54^. Chronic diastolic dysfunction results in the retrograde build-up of blood, with predominant accumulation in the lung^55^. As in the hypertrophied heart, the engorgement of tissue with blood results in weight gain. Increased lung weight is considered to represent blood congestion and implies diastolic HF^55^. In addition to anatomical differences, a notable symptom of HF is limited exercise capacity^22^. To assess locomotor ability in rodents, a treadmill or rotarod performance test is used^56,57^. Locomotor ability, however, can be affected by high brain function^58^. For this reason, decreased exercise capacity is not definitive but only supportive of the HFpEF phenotype.

### Animal model: comorbidity

To date, the treatment for HFpEF remains unclear^14^. A detailed study using an appropriate animal model enables drug development. Big data analysis has identified several comorbid factors: DM, obesity, hypertension, hyperlipidemia, and female sex^12,13^. Several established rodent models of HFpEF are listed in Table 1. Because of convenience, rodents are widely used to assess cardiac function and HF phenotype.

**Table 1:** Rodent models for human HFpEF research

Species	Comorbidity	Strain	Manipulation	Phenotypes
Rat	Hypertension	SHR		Hypertension and hypertrophy in 12 months HFpEF in 18 months
	Hypertension	Wistar	DOCA salt Unilateral nephrectomy 1% NaCl drinking water	Hypertrophy Severe hypertension
	Hypertension	DSS	4–8% NaCl chow	Severe hypertension Diastolic heart failure
Mouse	Hypertension	C57BL/6	DOCA salt Unilateral nephrectomy 1% NaCl drinking water	Mild hypertension Hypertrophy
	Hypertension	C57BL/6	Aldosterone Unilateral nephrectomy 1% NaCl drinking water	Hypertrophy Fibrosis Diastolic dysfunction
	Hypertension	C57BL/6	*Pressure overload* TAC AoB	Hypertrophy and fibrosis Diastolic dysfunction → systolic dysfunction
	Diabetes mellitus	*db/db*		Insulin resistance Hypertrophy Diastolic dysfunction
	Obesity	*ob/ob*		Hypertrophy Diastolic dysfunction
	Obesity + hypertension	C57BL/6	High-fat diet + L-NAME	Hypertrophy Diastolic dysfunction Pulmonary congestion
	Aging	SAMP		Diastolic dysfunction

The black arrow indicates the transition

*AoB* aortic banding, *DOCA* deoxycorticosterone acetate, *DSS* Dahl salt-sensitive, *L-NAME* L-NG-nitroarginine methyl ester, *SAMP* senescence-accelerated mouse prone 8, *SHR* spontaneous hypertensive rat, *TAC* transverse aortic constriction

Many research groups have used rats for hypertension research. Both systolic and diastolic pressures are sensitive to volume overload, the use of hypertensive agonists, and increased salt ingestion^59^. The spontaneously hypertensive rat (SHR) strain is widely used for human hypertension research^60^. Starting at <12 months of age, the SHR strain shows the gradual development of concentric hypertrophy with preserved contractility^60,61^. However, the strain develops cardiac decompensation, with left ventricular dilatation and reduced ejection fraction after 18 months^62^, a phenomenon rarely observed in human HFpEF. The SHR model closely resembles human HFpEF, but the phenotypic onset time is relatively late. It is difficult to maintain a group of these rats for ~1 year, making the use of the SHR strain expensive and time consuming.

In addition, the deoxycorticosterone acetate (DOCA)-salt model is an acceptable alternative for hypertension research^60^. Young adult Wistar rats (~8 weeks of age) undergo unilateral nephrectomy and are given drinking water with 1% sodium chloride. Starting 1 week after nephrectomy, the rats received subcutaneous injections of DOCA (25 mg in 0.4 mL of dimethylformamide every 4 days) for 28 days. The rats develop concentric hypertrophy, fibrosis, and hypertension^63^. Moreover, the DOCA-salt model can be used in mice, but blood pressure elevation is limited^64^. Thus, it is not clear whether the DOCA-salt model truly represents hypertension-induced HFpEF.

The Dahl salt-sensitive (DSS) rat with high-salt chow is considered a noninvasive and short-term HFpEF model. DSS rats fed 4–8% salty chow for 2 months develop typical HFpEF phenotypes^65,66^. The heart shows concentric hypertrophy, interstitial fibrosis, and diastolic dysfunction. The DSS with a high-salt diet model shows prolonged action potential, resulting in polymorphic ventricular tachyarrhythmia^65^. The DSS model, however, develops severe hypertension (>175 mmHg), which is uncommon in humans^67^. To accurately reflect human HFpEF, the DSS model would require the use of an antihypertensive drug.

Although rats are acceptable models for research on HFpEF, mice are easier to work with than rats. There is no significant change in blood pressure in mice compared with rats. Therefore, most acceptable hypertension models using rats are not comparable to those using mice. The ability to perform large-scale experiments with genetically modified animals and access to an animal bank is easier to accomplish with mice than with rats.

The DOCA model is applicable to both mice and rats^64^. DOCA salt combined with 1% sodium chloride causes hypertension in rats. Blood pressure is mildly increased or unchanged in mice, although concentric hypertrophy and interstitial fibrosis are observed. Unilateral nephrectomy in mice treated with DOCA salt leads to diastolic dysfunction. Unilateral nephrectomy was performed on C57BL/6 mice, followed by implantation of a DOCA pellet releasing 0.7 mg/day for 3 weeks; in addition, the mice were given 1% sodium chloride drinking water^68^. The DOCA model showed no difference in heart rate or ejection fraction. DOCA reduced exercise capacity, but pulmonary congestion was uncommon^69^.

In addition, in place of DOCA, infusion with d-aldosterone induces HFpEF^70^. Although applicable in rats, mice are considered more suitable for this model. C57BL/6 mice underwent implantation of d-aldosterone osmotic pumps (0.30 μg/h) after unilateral nephrectomy and were given 1% sodium chloride drinking water for 30 days^71^. Both systolic and diastolic pressure were elevated, but the heart rate was not changed. Moreover, systolic function was preserved. The mice developed concentric hypertrophy, diastolic dysfunction, pulmonary edema, and locomotor impairment^72^. Humans with HFpEF show high levels of aldosterone. The elevation of serum creatinine and albuminuria was detected in aldosterone model mice, similar to the findings in humans with HFpEF. Molecular events in the aldosterone infusion model were comparable to those in humans with HFpEF^4^. Hence, the aldosterone model represents both the clinical phenotype of HFpEF and the molecular events^73^.

Partial ligation or constriction of a large artery induces an abrupt and severe increase in afterload^74^. Constriction can be applied at the aortic root, transverse aorta, descending aorta, or even abdominal aorta^75^. The aortic root and transverse aorta are frequently selected. The constriction of a large artery generates a steep increase in blood pressure just proximal to the point of manipulation^76^. However, the segment distal to the constriction maintains normal blood pressure. Hence, the arterial constriction model resembles hypertension-based HFpEF, but the effect is limited to the heart. Aortic root constriction or aortic banding (AoB) usually results in eccentric hypertrophy due to intracardiac volume overload with excessive pulmonary edema^55^. Massive interstitial fibrosis accompanies hypertrophy, which interferes with ventricular movement. Although mice with AoB transiently maintain physical hemodynamics, they undergo aberrant decompensatory remodeling^77^. Prolonged AoB exacerbates systolic dysfunction, ultimately leading to HFrEF. The transition from HFpEF to HFrEF is rarely observed in humans^4^, and the findings after prolonged AoB do not correspond to clinical outcomes. Transverse aortic constriction (TAC) represents partial ligation between the brachiocephalic artery and left common carotid artery. TAC induces less severe vascular resistance and intraventricular volume overload than AoB due to the preservation of the brachiocephalic artery^76^. TAC induces concentric hypertrophy as well as eccentric hypertrophy with interstitial fibrosis. Moreover, prolonged TAC progresses to systolic heart failure. Hence, diastolic dysfunction induced by pressure overload fails to elicit human HFpEF^78^.

Since obesity and DM are notable comorbidities in HFpEF^79^, genetically modified *db/db*^80^ or *ob/ob*^81^ mice are widely used for cardiometabolic research. The *db/db* mouse has mutant leptin receptors that induce aberrant obesity. This leads to spontaneous insulin resistance, which is comparable to that in human type 2 DM. Without further administration of agonists, the *db/db* mouse shows diastolic dysfunction with concentric hypertrophy. The left ventricular ejection fraction remains in the normal range. Locomotor ability is associated with the severity of diastolic dysfunction^82^. The *ob/ob* mouse lacks leptins. Because of polyphagia, obesity and type 2 DM develop spontaneously. Disease progression in *ob/ob* mice resembles that in human type 2 DM. However, the use of the *ob/ob* mouse has some limitations. Leptin governs appetite control in addition to having a cardio-protective role. A loss of leptin impairs contraction-relaxation coupling in cardiomyocytes. Furthermore, leptin deficiency in human obesity is rare, meaning that the *ob/ob* mouse model is not appropriate for the study of human HFpEF^83^.

A noninvasive and nongenetically modified model of HFpEF was recently reported^84^. Schiattarella et al. considered common comorbidities in human HFpEF and created a model that combined hyperlipidemia and hypertension. They administered a high-fat diet with a nitric oxide synthase inhibitor ad libitum. After 5 weeks, they observed significant impairment of diastolic function, with pulmonary congestion and exercise intolerance. At 15 weeks, significant signs and symptoms of HFpEF had developed^84^. This model mimics human pathophysiology, implying its suitability for use in research.

In addition, age is an important risk factor for HFpEF^12^. The relative life span in rodents is even shorter than in humans, but “old” age in mice is only 18 months. It is difficult to maintain stable conditions during long-term rearing in a research facility. Therefore, the inbred senescence-accelerated mouse prone (SAMP) strain is useful to reduce the time course of aging^85^. Spontaneous acceleration of aging in the SAMP strain phenotype begins as early as postnatal 6 months. The SAMP8 strain shows cardiac hypertrophy with fibrosis as well as diastolic dysfunction. Systolic function is well preserved. The SAMP8 mouse carries more comorbidities associated with HFpEF than do other mice^86^. A detailed study using SAMP8 mice is required in the future.

## Future perspective and conclusion

Mortality and morbidity in HFpEF have continuously increased for more than two decades^3,4^. Furthermore, approximately half of newly diagnosed HF patients have preserved ejection fraction. The social cost of HFpEF has greatly increased, but no verified treatment regimen is available^4^. This brief review discussed the clinical trials and rodent models used for human HFpEF research. Disappointingly, several treatments that proved effective for HFrEF failed to improve the survival rate or even quality of life in HFpEF patients^9^. A limited number of Asian studies described the therapeutic potential of lipid-lowering statins, but the power of a randomized, multirace trial is required for verification^35^. In the TOPCAT study, the efficacy of spironolactone was unclear^30^. This mineralocorticoid receptor blocker reduced mortality only in patients enrolled in the United States, implying that genetic background could be a confounding factor in HFpEF. Detailed and homogeneous grouping of HFpEF patients could resolve this question.

Rats and mice have been used for the study of human HFpEF. Most rodent HFpEF models show concentric or eccentric hypertrophy, interstitial fibrosis, and diastolic dysfunction. For the assessment of underlying molecular events, vascular inflammation, endothelial dysfunction, or microvascular uncoupling was suggested^47^. Cumulative data revealed that pharmacological intervention for cardiac hypertrophy ameliorates cardiac fibrosis accompanying the ventricular stiffness that results in diastolic dysfunction^87^. Histone deacetylase (HDAC) inhibitors are considered promising for the treatment of cardiac hypertrophy and therefore diastolic dysfunction^50,53,88–90^. In a rodent model, cardiac hypertrophy was completely blocked when HDAC inhibitors were concurrently administered^50,88–90^. Moreover, our group showed that HDAC inhibitors could induce the regression of preexisting hypertrophy and fibrosis^91^. HDAC inhibitors should be considered novel therapeutic agents for HFpEF. HDAC inhibitors were first developed as an anticancer agent, which suggests that using HDAC inhibitors as HFpEF modulators may have cytotoxic side effects. In fact, notable cytotoxicity-associated adverse reactions of HDAC inhibitors such as SAHA include hair loss, diarrhea, peripheral numbness, or tingling sensation^92^. Furthermore, our group demonstrated vascular calcification as a potential side effect of HDAC inhibitors^93^. Since DM is one of the notable comorbidities of HFpEF, using HDAC inhibitors to treat vulnerable patients should be avoided. In addition, immune modulators may be useful in HFpEF. Hulsmans et al. reported the contribution of macrophages to diastolic dysfunction^94^. In fact, a clinical trial using interleukin-1β, the Interleukin-1 Blockade in Heart Failure with Preserved Ejection Fraction (HFpEF): A Randomized Placebo-controlled Double-Blinded Study (D-HART2), has been completed and the results have been reported^95^. While systemic inflammation was significantly improved, survival benefits were not observed. The power of the D-HART2, however, was relatively small, which was a weak point of the study^95^. Thus, another immune modulator should be considered. Endothelial dysfunction failed to produce nitric oxide or improvement in vascular elasticity. Restoration of either endothelial nitric oxide synthase activity or cGMP activity was attempted. Organic nitrate donors and inorganic nitrate suppliers showed no benefit for exercise intolerance^23,33^.

Physicians have focused on symptom control due to the lack of therapeutic agents^3,4^. Numerous clinical trials have been instituted, but these require long-term follow-up. To correctly understand HFpEF, an animal study of HFpEF should be conducted in parallel with a human study. Promising outcomes from animal research should be considered when developing treatment regimens in humans. Combined comorbidity is common in humans with HFpEF, making the development of a rodent model difficult. It is noteworthy that a rodent model with more than two comorbidities in a single mouse has been generated^84^. Nonetheless, understanding HFpEF in a rodent model could aid in the understanding of human HFpEF.
